# A systematic review of sub-microscopic *Plasmodium vivax* infection

**DOI:** 10.1186/s12936-015-0884-z

**Published:** 2015-09-22

**Authors:** Clarissa M. Moreira, Mahmoud Abo-Shehada, Ric N. Price, Chris J. Drakeley

**Affiliations:** WorldWide Antimalarial Resistance Network (WWARN), Oxford, UK; Nuffield Department of Medicine, Centre for Tropical Medicine and Global Health, Oxford University, Oxford, UK; Faculty of Epidemiology and Population Health, London School of Hygiene and Tropical Medicine, University of London, London, UK; Gobal and Tropical Health Division, Menzies School of Health Research, Charles Darwin University, Darwin, Australia; Department of Immunology and Infection, London School of Hygiene and Tropical Medicine, London, UK

**Keywords:** Malaria, *Plasmodium vivax*, Polymerase chain reaction, Microscopy, Diagnostics, Prevalence

## Abstract

**Background:**

An accurate estimate of *Plasmodiumvivax* prevalence is essential for the successful implementation of malaria control and elimination programmes. Prevalence estimates both inform control strategies and are used in their evaluation. Light microscopy is the main method for detecting *Plasmodium* parasitaemia in the peripheral blood, but compared to molecular diagnostics, such as polymerase chain reaction (PCR), has limited sensitivity.

**Methods:**

A systematic review and meta-analysis was conducted to assess the effect of detection method on the prevalence of *P.vivax* and to quantify the extent to which *P. vivax* infections are undetected by microscopy. Embase, Medline and the Cochrane Database were searched for studies reporting prevalence by PCR and by microscopy and that contained all of the following key words: *vivax*, PCR, and malaria. Prevalence estimates and study meta-data were extracted systematically from each publication. Combined microscopy:PCR prevalence ratios were estimated by random effects meta-analysis. Sensitivity and specificity of microscopy were calculated using PCR as the gold standard.

**Results:**

Of 874 studies reviewed, 40 met the criteria for inclusion contributing 54 prevalence pairs. The prevalence of *P.vivax* infection measured by PCR was consistently higher than the prevalence measured by microscopy with sub-patent parasitaemia. The mean prevalence of infection detected by microscopy was 67 % (95 % CI 59–73 %) lower than the prevalence detected by PCR. The detection of sub-patent parasitaemia did not vary according to the microscopy method (thick or, thick and thin smears), the PCR prevalence (as a measure of the true *P.vivax* prevalence), the type of blood used or DNA extraction method.

**Conclusions:**

Quantifying *P. vivax* parasitaemia by PCR rather than microscopy consistently increased prevalence estimates by a factor of 2.3. Whilst the sensitivity of microscopy can be improved by better methods, molecular methods have potential to be scaled up to improve the detection of *P. vivax* transmission reservoirs.

**Electronic supplementary material:**

The online version of this article (doi:10.1186/s12936-015-0884-z) contains supplementary material, which is available to authorized users.

## Background

Almost half of the world’s population is at risk of infection with *Plasmodium vivax* [1]. Most cases originate in South East Asia and the Western Pacific and a significant number occur in South America and the Horn of Africa [1, 2]. *P. vivax* is associated with substantial morbidity and severe and fatal disease in endemic countries [3–6]. *P vivax* infection is characterized by comparatively low parasitaemia compared to *Plasmodiumfalciparum,* and in co-endemic regions vivax parasitaemia is often conservatively documented as *P. falciparum* reducing the reported prevalence [3]. Many nations evaluating their prospects for malaria elimination are endemic for *P. vivax* and as successful control programmes reduce the risk of *P.falciparum* the relative proportion of *P.vivax* infection rises. Accurate data on *P.vivax* prevalence and transmission patterns is important for the progress of elimination campaigns and focusing malaria control efforts [4].

Light microscopy examination of blood films is the main method for detecting peripheral parasitaemia and for differentiation of *Plasmodium* species [5]. The World Health Organization (WHO) guidelines for malaria diagnosis and treatment recommend that malaria treatment be given only after a positive parasitological test result from either microscopy or a rapid diagnostic test (RDT) [6]. The advantages of light microscopy make it an ideal diagnosis tool in resource poor settings however its accuracy depends upon the technician’s skill level and can be adversely affected by operational constraints or technical problems. Low-density infections often remain undetected by microscopy [7, 8]. As *P*. *vivax* is characterized by low level parasitaemia microscopy may not be the most appropriate tool for accurate diagnosis. Molecular methods, e.g. polymerase chain reaction (PCR), depend on DNA amplification approaches and have higher sensitivity than microscopy [9]. Despite the greater sensitivity of PCR it is not widely used due to the lack of a standardized methodology, high costs, and the need for highly-trained staff. PCR is increasingly used in epidemiological studies, but rarely used in routine clinical diagnosis.

Most *P.vivax* cases occur in low transmission settings where asymptomatic carriage occurs relatively frequently [10]. With the reliance on symptoms driving the presentation of infected patients and microscopy used as the main diagnosis tool, asymptomatic and sub-microscopic infections are likely to remain undetected and untreated in vivax endemic populations. Sub-microscopic infections of *P. falciparum* have been shown to infect mosquitoes and transmit malaria [11]. Sub-microscopic *P. vivax* infections may similarly contribute to the infectious reservoir and maintain transmission. A review on asymptomatic malaria in Brazil highlighted the importance of accurate diagnosis and detection of all *Plasmodium* cases for the malaria control programmes to be effective [7]. A prevalence ratio reported from a study in Peru indicates that 78 % of infections would go undetected if microscopy alone was used in surveillance programmes [8]. In a study from Iran microscopy did not detect any vivax infection in the 900 samples, whereas PCR detected ten positive samples out of 871 [12].

In a review exploring the proportion of sub-microscopic *P. falciparum* infections, Okell et al. documented reduced sensitivity of microscopy which detected on average 50 % of falciparum infections detected by PCR [13]. Cheng et al. recently reviewed 25 publications (31 surveys) from 1996 to 2010 reporting a mean of 69.5 % sub-microscopic *P.vivax* infections [14]. The current review extends the work of Cheng et al. with a meta-analysis and the inclusion of additional studies; since 2010, a further 11 prevalence studies reported microscopy and PCR *P. vivax* prevalence and an additional seven were identified from 1996 to 2010. The aim was to re-quantify the extent of sub-microscopic *P. vivax* infections detected by PCR and identify whether this ratio varies with transmission intensity, location of study or laboratory methods.

## Methods

This systematic review used a predefined protocol and followed the PRISMA guidelines [15]. PubMed, EMBASE and the Cochrane Library were searched using MeSH search terms and Boolean operators: “malaria” AND “PCR” AND “vivax” up to the 15 September 2014. The search was restricted to English language publications with no time limits. Results of each search were exported and duplicates removed. This high-yield search strategy was used to ensure capture of all relevant articles. Titles and abstracts of all articles were initially scanned to identify prevalence studies. Case reports, case series, efficacy studies, entomological surveys, non-human studies, immunological studies, genetic sequencing studies and other non-relevant articles were excluded. Full texts of articles identified as potentially relevant in this initial screen were assessed against the full eligibility criteria using a standardized form. References of relevant articles were scanned to identify additional studies.

### Selection criteria

Eligible studies reported the prevalence of *P. vivax* by microscopy and PCR in the same population living in a malaria endemic country. If only a subset of participants were tested by microscopy or PCR, the study was included providing the subset was selected randomly. Exclusion criteria included: studies of imported malaria, studies where a large-scale intervention was implemented before the measurement of prevalence (e.g. insecticide-treated bed nets or mass drug administration), studies in pregnant women, studies that select participants based on parasitaemia levels, malaria symptoms or malaria diagnosis, studies of a population not representative of a defined endemic area (e.g. studies in refugees or migrant workers), studies with less than 20 blood samples tested by either method, and studies where no *P. vivax* was detected by PCR or microscopy. When the presence of malaria symptoms was not specifically stated it was assumed that participants seeking treatment or care at health facilities were symptomatic and these studies were excluded. In cohort studies, data were extracted from the baseline observation only.

### Data extraction

From each eligible study the following information was extracted: month and year of sample collection, location, age criteria for inclusion, age range of included participants, prevalence of *P. vivax* malaria by microscopy, prevalence of *P. vivax* malaria by PCR, number of false-positives (i.e. number of samples microscopically positive and PCR negative) and number of false-negatives (i.e. number of samples microscopically negative and PCR positive) and PCR and microscopy methodology. Authors were not contacted for further information and no studies were excluded on the basis of quality.

### Statistical analysis

The parasite prevalence by microscopy and PCR were calculated for each study, once for *P.vivax* monoinfections and again for all *P.vivax* infections (i.e. infections where *P.vivax* was detected either alone or with any other *Plasmodium* species). Prevalence of infection detected by microscopy was compared to the prevalence of infection detected by PCR to produce a microscopy:PCR prevalence ratio and the sub-microscopic prevalence was calculated as: PCR prevalence minus the microscopy prevalence. PCR was considered the reference standard and the numbers of false-positives and false-negatives were used to calculate sensitivity and specificity estimates for microscopy when this information was available. Log transformed microscopy: PCR prevalence ratios and the sample size for each study were used to derive inverse-variance weighted fixed effects and random effects meta-analysis combined estimates [16]. The Kruskal–Wallis method was used for nonparametric comparisons, and Student’s *t* test for parametric comparisons. For categorical variables percentages and corresponding 95 % confidence intervals (95 % CI) were calculated using Wilson’s method. Proportions were examined using χ^2^ with Yates correction or by Fisher’s exact test. The Chi squared heterogeneity statistic was used to assess the between-study heterogeneity. Forest plots and combined random-effects estimates were produced for sub groups (e.g. microscopy method) to determine if heterogeneity was accounted for by any methodological differences. All analyses were performed using Stata software (11.0; StataCorp).

## Results

### Literature search

**Fig. 1:**
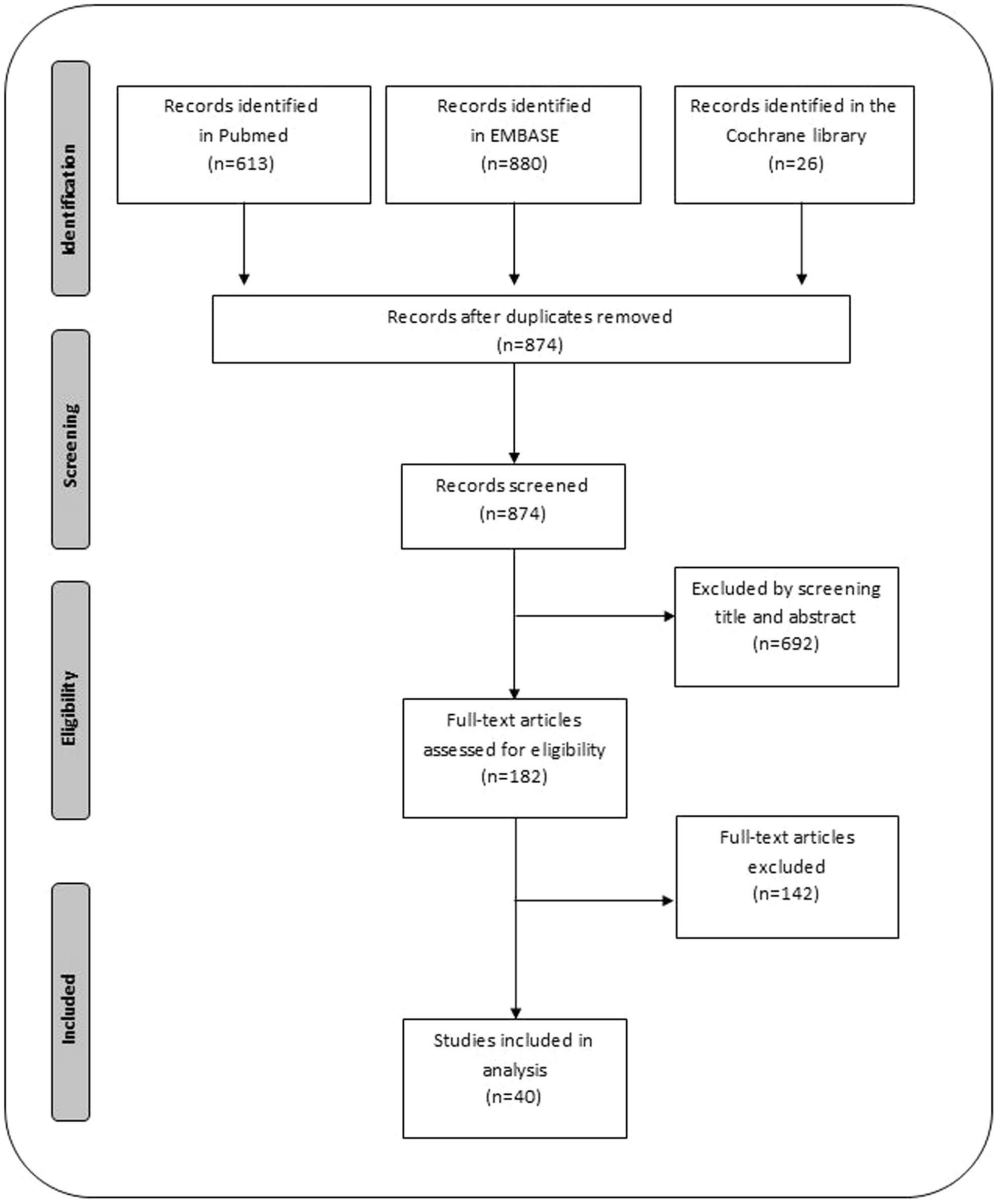
PRISM flow diagram. Study selection (PRISM flow diagram)

A search of Pubmed, EMBASE and the Cochrane Library identified 874 unique publications; 182 were selected for full text evaluation. Forty met all eligibility criteria (Fig. 1) [8, 12, 17–54]. Details for the exclusion of full text articles are provided in Additional file 1. The most common reasons for exclusion were surveys of patients with malaria or malaria symptoms (N = 84), study was not population based (N = 24), prevalence from either method was not reported (N = 17) and samples chosen for PCR were not randomly selected (N = 8). Five studies were excluded since no *P.vivax* parasites were detected either by PCR or by microscopy [55–59].

### Description of included studies

Most of the 40 included studies were derived from cross–sectional surveys (N = 31, 78 %), three were cohort studies [23, 47, 60] and three studies recruited participants via active case detection [30, 44, 46]. Three studies did not report the study design [23, 51, 61].

The sampling strategy was reported for 27 studies (68 %). In 14 (52 %) of these studies, all households were visited and informed consent sought from all eligible residents. Five studies reported random sampling of individuals, two studies reported random sampling at the household level, one reported random sampling of schools where all students at the selected schools were included and one study reported multistage cluster sample with random cluster sampling of villages then random household sampling. Two studies reported convenience sampling (one of which provided no further information [36]) and the other took blood samples from all neighbours of confirmed malaria cases admitted to hospital [20]. Two studies reported active case detection, one at mobile malaria clinics [26] and the other through contact tracing of confirmed malaria cases and high risk group screening [42].

The 40 publications yielded 54 pairs of microscopy and PCR prevalence estimates. In 14 studies prevalence was reported for different localities which were analysed as independent observations. Two studies compared microscopy to two different PCR methods contributing two paired prevalence estimates [47, 62]. Key study features are highlighted in Additional file 2. Most prevalence estimates were from Papua New Guinea (N = 13) and Brazil (N = 13). Only two prevalence pairs were from Africa and one from the Middle East. Blood samples were collected between 1996 and 2012, with the majority collected after 2004 (N = 34). Eight studies did not report the year of sample collection. Most blood samples were from participants of all ages, five studies were restricted to children only (maximum age 3 or 5 years or age limited not reported), two restricted to adults aged more than 15 years and four studies did not report the age of participants (see Additional file 2).

In 73 % (29/40) of studies all blood samples were assessed by both microscopy and PCR. In five studies more than 90 % of microscopy samples were assessed by PCR [17, 21, 23, 28, 37]. For three prevalence estimates, 8, 19 and 34 % of microscopy samples were randomly selected for molecular testing [36, 37]. One study tested 85 % of microscopy samples by PCR but did not report how these samples were chosen [20], another tested 80 % of samples by PCR due to a reported difficulty in collecting whole blood samples from children less than 5 years [8]. Another study selected all samples from villages where there was any microscopy-positive sample, plus a 10 % random selection of microscopy-negative samples from all villages for testing by PCR [18]. In this last case, the majority of samples tested by PCR were not samples that were confirmed microscopy-positive (only 8 samples out of 3223 tested by PCR were chosen because they were positive by microscopy), so this study was included. The final study selected 7 % of microscopy samples that were chosen to be representative of the age, gender and village composition for testing by PCR [61].

The overall median sample size was 504 (range 79–16,229) for microscopy and 482 (range 79–8590) for PCR. Most microscopy measurements used thick and thin smears with Giemsa staining (N = 28), the remaining studies (N = 12) used only thick smears with Giemsa staining. The most referenced PCR method was nested PCR using the protocol of Snounou et al. (N = 18) [9]. Other reported PCR methods include the semi-quantitative PCR (LDR-FMA) of McNamara, the semi-nested multiplex PCR of Rubio, various real-time PCR methods [22, 45, 62, 63] and one article reported using a newly designed mitochondrial-DNA-based PCR method [28].

All studies described the type of blood and DNA extraction method used for PCR analysis. Twenty studies (50 %) used whole blood (primarily >200 µL, N = 15), 17 (42.5 %) used blood from filter paper, and three studies (7.5 %) reported using both blood from filter paper and whole blood for DNA extraction. Two of the three studies that reported both methods used filter paper for some surveys and whole blood for others and the other study used whole blood for participants aged >5 years and filter paper for participants <5 years.

### Quality assessment

Only four studies reported blinding of technicians to results of the other detection method (i.e. microscopists blinded to PCR results and vice versa). Double reading of microscopy slides was reported for 40 prevalence pairs (74 %). Many studies reported the use of ‘expert’ microscopists and specified their training, years of experience or results when given a blinded test before the study started. Only seven studies reported blinding of the second microscopists to the results of the first, most did not report if this occurred (N = 30). Nineteen studies (48 %) reported the use of negative controls during PCR.

### Prevalence measurements

**Fig. 2:**
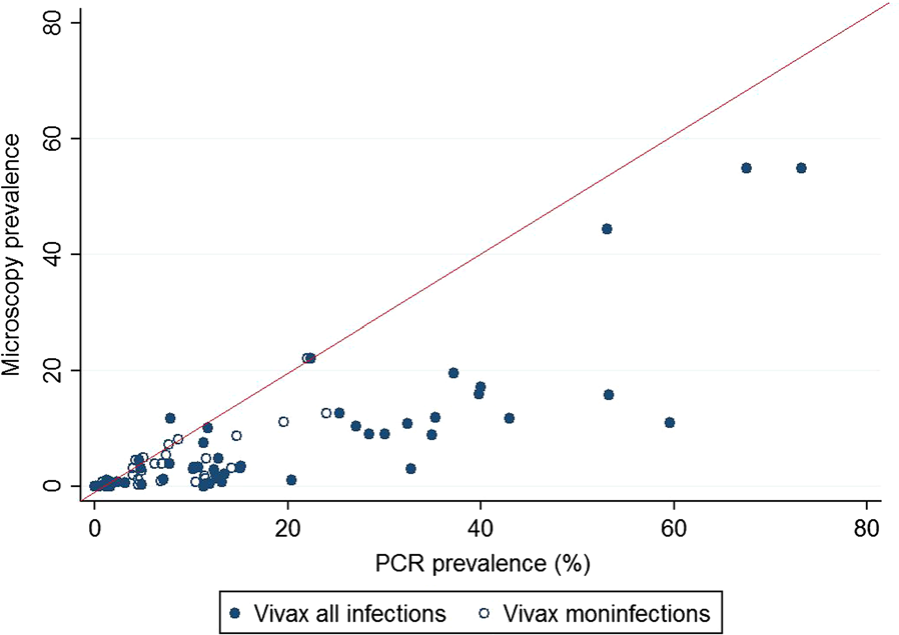
PCR *P.* *vivax* prevalence versus microscopy *P.* *vivax* prevalence, monoinfections and mixed infections. *Scatter plot* of the prevalence of *P.* *vivax* infection as determined by PCR versus prevalence of *P. vivax* as detected by microscopy. Prevalence pairs including only monoinfections (infections with only *P. vivax*) are shown in *open circles* and the prevalence pairs including all infections (*P. vivax* monoinfections and *P. vivax* infections where another species is also detected) are shown in *filled circles*. The *line* shows the expected association if both techniques were equally sensitive

**Fig. 3:**
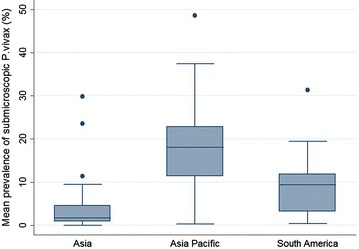
Sub-microscopic *P. vivax* prevalence by region. *Box plots* showing the median and the IQR of sub-microscopic *P. vivax* prevalence by region (*Asia* Thailand, Sri Lanka, Lao PDR, Vietnam, Malaysia and Cambodia; *Asia Pacific* Indonesia and Papua New Guinea; *South America* Brazil, Venezuela and Peru)

The prevalence of *P*. *vivax* infection measured by PCR was consistently higher than the prevalence measured by microscopy (Fig. 2). One study reported a prevalence by microscopy higher than the prevalence by PCR [39]. The mean prevalence by PCR was 18 % (95 % CI 13.3–23.4) and the mean prevalence by microscopy was 7.8 % (95 % CI 4.4–11.1). Forty-two of the 54 prevalence pairs reported the prevalence of *P.vivax* monoinfections (infection with *P. vivax* only) separately from the prevalence of all *P.vivax* infections (mono or mixed infections). The variation in prevalence was greater when including mixed infections compared to including monoinfections only, i.e. concordance between the two techniques was greater when considering monoinfections only (Fig. 2). PCR detected mixed infections in 1.8 % of parasitaemias compared to 0.65 % detected by microscopy. For nine prevalence pairs, *P. vivax* parasitaemia was not detected by microscopy but was detected by PCR (PCR prevalence range 0.18–11.2 %). The prevalence of sub-microscopic *P. vivax* ranged from 0.01 to 48.8 % and was higher in the Asia Pacific region [mean = 18 % (range 0.32–48.8 %)] compared to South America [9.4 % (range 0.34–31.3 %); p = 0.02] and Asia [1.7 % (range 0.01–29.8 %); p = 0.001]. There was no statistical difference in the prevalence of sub-microscopic *P. vivax* between Asia and South America (p = 0.18; Fig. 3).

### Prevalence ratios

**Fig. 4:**
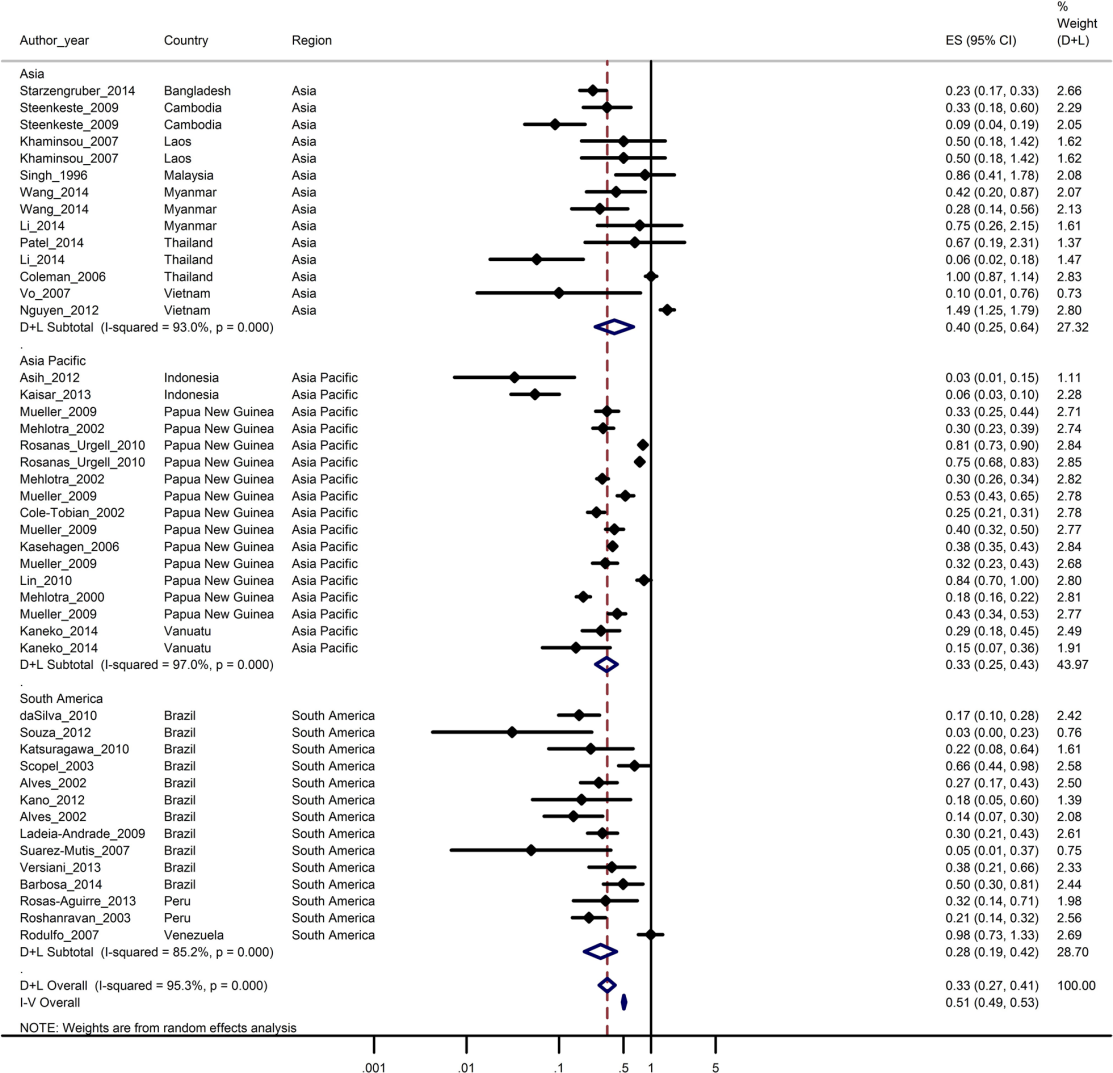
Ratios of the prevalence of *P. vivax* determined by microscopy to that determined by PCR. *Forest plot* displaying ratios (represented as *closed diamonds*) and 95 % confidence intervals (*horizontal lines*) of the prevalence of *P. vivax* detected by microscopy compared to that detected by PCR in 36 pair observations. The *open diamond* and *broken vertical line* represents the combined estimate from a random effects (D + L overall) meta-analysis, the combined estimate from a fixed effect (I − V overall) is also shown. The *unbroken vertical line* is at null value (1)

Prevalence ratios were calculated for 83 % (45/54) of the prevalence pairs; in the remaining nine pairs no *P.vivax* was detected by microscopy precluding derivation of a ratio. A forest plot of the prevalence ratios with 95 % confidence intervals is shown in Fig. 4. The prevalence ratios were highly heterogeneous between studies and ranged from 0.03 to 1.49 (heterogeneity Chi squared = 936.51, p < 0.001, Fig. 4). The combined microscopy: PCR prevalence ratio estimate using random effects at the study level was 0.33 (95 % CI 0.27–0.41), i.e. the prevalence of infection detected by microscopy was 67 % lower than the prevalence of infection detected by PCR. The corresponding figure when considering monoinfections only, was 0.37 (95 % CI 0.29–0.48). Overall the prevalence of infection detected by microscopy was 60 % (95 % CI 36–75 %) lower than the prevalence of infection detected by PCR in Asia, 67 % (95 % CI 57–75 %) lower in the Asia Pacific and 72 % (95 % CI 58–81 %) lower in South America. Studies from South America were less heterogeneous (heterogeneity Chi squared = 88.03) compared to Asia (185.07) and Asia Pacific (88.03).

**Fig. 5:**
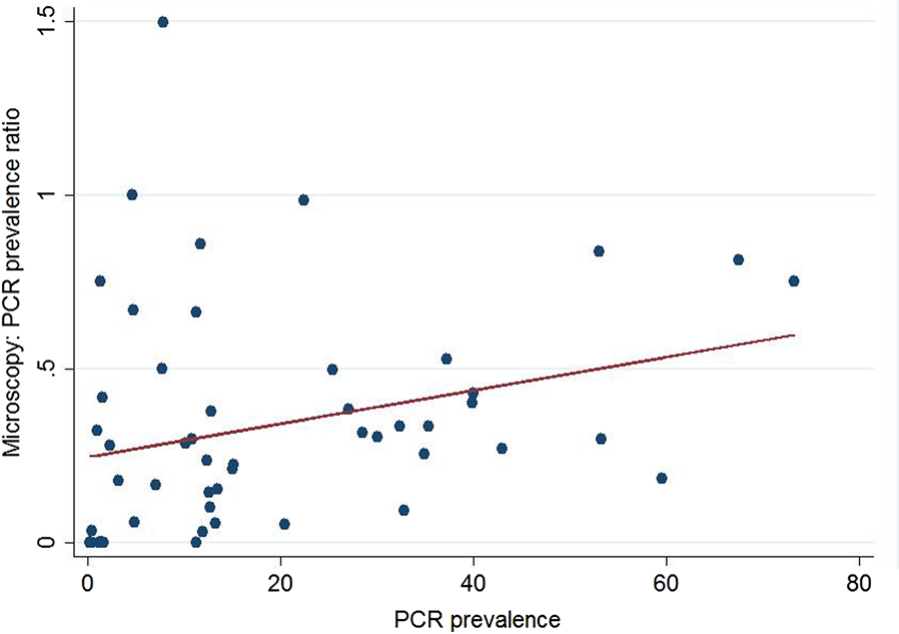
*Scatter plot* of the microscopy: PCR prevalence ratio versus the underlying PCR prevalence

The combined prevalence ratio estimates did not vary between studies that used thick and thin smears for microscopy and those that used thick smears only. Likewise the prevalence ratio did not vary between studies that used filter paper for DNA extraction compared to those that used whole blood. However the majority of prevalence pairs (14/23) that used whole blood were from Asia, whereas prevalence pairs that reported filter paper for DNA extraction were from the Asia Pacific region (14/28) or South America (10/28). Reporting the use of a negative control was not associated with a significant difference in the combined microscopy: PCR prevalence ratio: 0.36 (95 % CI 0.27–0.47) versus 0.31 (95 % CI 0.23–0.42), (p = 0.24). Prevalence ratios did not significantly vary between studies that used nested PCR compared to other PCR techniques (p = 0.71). The microscopy: PCR prevalence ratio showed a weak positive association with the underlying PCR prevalence, increasing by 0.047 (95 % CI 0.002–0.09) per 10 % increase in PCR prevalence (Fig. 5).

### Sensitivity and specificity

The sensitivity and specificity of microscopy was calculated for 20 prevalence paired estimates. Sensitivity and specificity were 0 and 100 % respectively for 7 prevalence pairs where no *P.vivax* parasites were detected by microscopy. Assuming that one sample was positive by both microscopy and PCR, and that one sample was positive by microscopy and negative by PCR allowed calculation of sensitivity and specificity for these seven studies; results are available in Additional file 3. Overall, microscopy was highly specific with less than 2 % false positives. However the sensitivity varied from 4.2 to 94 %.

## Discussion

Data from 40 published studies show that PCR detects 67 % more *P.vivax* infections than microscopy in surveys of endemic populations. This result is similar to findings by Cheng et al. who reported on 25 studies in which *P.vivax* was not detected by microscopy in 69.5 % of estimates. The additional 18 studies included in the current analysis generally reported higher prevalence by both microscopy and PCR; the mean PCR prevalence was 18.6 % (range 0.18–73.2 %) compared to a range of PCR prevalence estimates of 0.2–59.5 % reported by Cheng.

Both reviews are also consistent with a literature review of *P. falciparum* PCR and microscopy prevalence reported by Okell and colleagues in which microscopy detected 51 % of all parasitaemias detected by PCR [13]. There are several characteristics of *P.vivax* infections that make diagnosis by microscopy more difficult than diagnosis of *P. falciparum* including the fact that *P*. *vivax* and *P. falciparum* are co-endemic in most vivax endemic areas and the detection of lower levels of *P. vivax* parasitaemia is difficult in the presence of higher levels of *P. falciparum* parasitaemia [3]. PCR has greater sensitivity to identify mixed infections [64], and this was apparent in the discrepancy between microscopy and PCR being greatest when considering all *P. vivax* infections (mono and mixed infections). Similar results are apparent in cross sectional surveys where PCR is as much as 30-fold more efficient at detecting mixed infections than microscopy alone [17]. The difference between prevalence ratios when detecting monoinfection and prevalence ratios when detecting all *P.vivax* infections, showed a similar trend although this did not reach statistically significance likely due to the limited number of prevalence pairs.

Only ten studies reported sensitivity and specificity measurements and these studies specifically aimed to measure these parameters for either PCR or microscopy. The wide range of microscopy sensitivities mirrored published sensitivities although microscopy sensitivity <50 % is less frequently reported in the literature than observed here. The sensitivity and specificity of microscopy is highly dependent on slide quality and the skill of microscopists [65]. Unfortunately these factors could not be considered in this analysis; both are subjective and rarely reported. The low prevalence of vivax malaria could explain the variability in sensitivity estimates as the number of positive results for both PCR and microscopy used in sensitivity calculations was small.

The studies included in this review are representative of the geographical distribution of *P. vivax,* with most but not all *P.vivax* endemic countries represented. The inclusion of only English publications introduced a bias towards studies from English speaking countries and a large number of potentially eligible publications from Brazil were not available in English [66]. The analysis focused on blood samples representative of endemic populations with a high proportion of included studies adopting comprehensive sampling strategies in an attempt to determine covariates in endemic populations; however the detection of sub-microscopic infections in high-risk groups or other populations excluded from this review also have public health relevance that warrants investigation.

With only 54 paired prevalence estimates included in this meta-analysis, the power to detect key covariates was limited. Studies where patients were selected on the basis of malaria symptoms were excluded and this one criterion significantly reduced the number of studies available. Five studies were excluded as no *P. vivax* was detected by either technique, in addition, prevalence ratio could not be calculated for nine prevalence pairs when no *P. vivax* was detected by microscopy, the least sensitive detection method. Therefore, the extent to which microscopy failed to detect infections of *P*. *vivax* in low transmission areas is likely underestimated. Sample size is an important consideration in the design of studies where few infections are detected. The studies in this review that did not microscopically detect any *P. vivax* infections but did detect vivax infections with PCR were of varying samples sizes and two were large studies (N = 3316, N = 1527). This indicates that when only using microscopy, increasing the sample size alone will not allow detection of all existing *P. vivax* infections.

The detection limit of both techniques is dependent on the volume of blood examined. In theory, less blood is examined using standard microscopy techniques (~5 µL) than when a PCR assay is used [60, 67]. This meta-analysis did not consider the exact volume of blood examined since the amount of blood used for DNA extraction for PCR was reported in only 18/40 studies and the amount of blood taken from participants reported in only 5/40 studies. Furthermore, nearly all studies that did report the amount of blood used for DNA extraction reported using 200 µL. The type of blood sample and DNA extraction method used for PCR analysis was examined and while the PCR prevalence was higher in studies that used whole blood versus studies that used filter paper this likely reflected regional variation and since the majority of studies using whole blood were from Asia and the majority of studies using filter paper being from the Asia Pacific or South America.

Future studies should examine the prevalence ratios of all malaria species in the same population to see if the sensitivity and specificity of microscopy and PCR varies according to the species examined. Although PCR is unlikely to be routinely used for screening in low-income countries, quantification of sub-microscopic infections under different transmission settings could be used to accurately estimate the true prevalence of *P.vivax* infections. Mathematical models could predict the extent of sub microscopic infections given a number of key parameters on the population characteristics and the transmission setting. Bayesian approaches have already been used to estimate disease prevalence in the absence of a gold standard diagnostic test or when the gold standard has imperfect sensitivity and specificity [68]. The current review has identified gaps in the information required for mathematical models designed to accurately estimate prevalence of *P. vivax* infection. Health planning and decision making by malaria control and elimination programmes in *P.vivax* endemic areas will require reliable estimates of parasite prevalence. The results of this review highlight the benefits of investing in PCR techniques to inform malaria control programmes. In areas focused on elimination it is vital that all reservoirs of *P.vivax* malaria are detected especially since rates of asymptomatic carriage can be substantial with a known ability to transmit [69].

## Additional files

**Additional file 1.** Studies excluded following evaluation of full text (N = 142) according to the main reason for exclusion.
**Additional file 2.** Summary of study characteristics.
**Additional file 3.** Sensitivity and specificity of microscopy compared to PCR.
