# Supplementary material for: A systematic review of sub-microscopic Plasmodium vivax infection
Source: Malar J. 2015 Sep 22;14:360. doi: 10.1186/s12936-015-0884-z (PMC4578340; doi:10.1186/s12936-015-0884-z)
Supplement: Supplementary file 1 — Additional file 1. Studies excluded following evaluation of full text (N = 142) according to the main reason for exclusion. [file 12936_2015_884_MOESM1_ESM.docx]

**Studies excluded following evaluation of full text (N=142) according to the main reason for exclusion**

| **Reason** | **Excluded studies (first author & year)** |
| --- | --- |
| Conference abstract only, full study details not available | Villasis 2009[1], Dacuma 2011[2], Alencar 2011[3], Soto-Calle 2010[4] |
| Data on *vivax* pooled with other *plasmodium* species | Ali 2005[5], Manning 2013[6], Khan 2014[7] |
| Full text not available | Felger 1995[8], Lee 1999[9] |
| Microscopy and PCR prevalence not reported separately | Pacific Malaria Initiative Survey Group 2010[10], Khan 2011[11], Rodulfo 2007[12], Steenkeste 2010[13], Andrade 2011[14], Andrade 2009[15], Carrara 2013[16] |
| No parasitemia detected by either method | Das 2005[17], de Almeida 2010[18], Gomes 2008[19], Turki 2012[20], Zoghi 2012[20] |
| Pregnant women only included | Agudelo 2013[21] |
| Not population based | Mens 2012[22], Arai 1996[23] |
| Not population based - migrant workers | Kritsiriwuthinan 2011[24] |
| Not population based - soldiers deployed to endemic area | Kain 1993[25], Brown 1992[26] |
| Not population based - blood donors | Contreras 2011[27], Arroyo Sanchez 2011[28], Chaijaroenkul 2011[29] |
| Not population based - hospital inpatients | Santana-Morales 2012[30] |
| Not population based - refugees | Monge-Maillo 2011[31], Lindo 2007[32], Matisz 2011[33], Ndao 2004[34] |
| Population chosen on the basis of malaria symptoms | Osman 2010[35], Bernabeu 2012[36], Rougemont 2004[37], Bendezu 2010[38], Iqbal 1999[39], Veron 2009[40], Pöschl 2010[41], Heidari 2009[42], Cnops 2010[43], Andrade 2010[44], Cheng 2013[45], Proux 2011[46], Bharti 2013[47], Eibach 2013[48], Parajuli 2009[49], Noteghpour 2011[50], Zakeri 2004[51], Zakeri 2002[52], Yan 2013[53], Woldearegai 2013[54] , Johnston 2006[55], Woyessa 2013[56], Kim 2010[57], Maltha 2010[58], Boonma 2007[59], Postigo 1998[60], Joveen-Neoh 2011[61], Auma 2013[62], Mohon 2012[63], Gargate 2009[64], Orjih 2008[65], Snounou 1993[66], Hwang 2011[67], Mohapatra 2008[68], Parkes 2001[69], Fuehrer 2010[70], Kuamsab 2012[71], Pakalapati 2013b[72], Barman 2003[73], Mens 2008[74], Barker 1994[75], Lekweiry 2009[76], Noedl 2006[77], Pakalapati 2013a[78], Haghdoost 2006[79], Laoboonchai 2001[80], Tao 2011[81], A-Elgayoum 2010[82], Alam 2011[83], Genc 2010[84], Ebrahimzadeh 2007[85], Lekweiry 2011[86], Laserson 1994[87], Swan 2005[88], Baltzell 2013[89], Singh 2010[90], Zhou 2013[91], Alemu 2013[92], Puri 2013[93], Mekonnen 2014[94], Herrera 2014 [95], Najafabadi 2014(1)[96], Najafabadi 2014(2)[97], Singh 2014[98], Alemu 2014 [99], Zhou 2014 [100], Kim 2014[101], Shahzadi 2013(1)[102], Shahzadi 2013(2)[103] |
| Population is confirmed malaria cases | Ayala 2006[104], Tirasophon 1995[105], Maltha 2012[58], Han 2007[106], Kho 2003[107], Zakeri 2010[108], Gupta 2010[109], Van den Eede 2011[110], Farooq 2009[111], Reller 2013[112], Putaporntip 2011[113], Dormond 2011[114], Ramírez-Olivenci 2012[115], Congpuong 2008[116], Albuquerque 2009[117] |
| Prevalence by PCR not reported | Lek 2010[118], Owusu-Agyei 2002[119], Kalayjian2013[120], Sattabongkot 2014[121] |
| Prevalence by microscopy not reported | Noor 2011[122], Ataka 2001[123], Cucunubá 2013[124], Canier 2013[125], Mendes 2011[126], Fru-Cho 2014[127] |
| Reported in Asih 2012 | Herdiana 2013[128] |
| Reported in Cucunuba 2013 | Cucunubá 2008[129] |
| Reported in DaSilva 2008 | da Silva-Nunes 2008[130] |
| Reported in Kasehagen 2006 | Kasehagen 2007[131] |
| Reported in Nguygen 2012 | Thang 2009[132] |
| Reported in Scopel 2004 | Scopel 2004[133] |
| Review of other articles | Culleton 2008[134] |
| Samples tested by PCR not randomly chosen | Harris 2010[135], Van Den Eede 2009[136], Win 2002[137], Bharti 2007[138], Quintana 1998[139], Dawoud 2008[140], Mahajan 2012[141], Curado 2006[142] |

1. Villasis E, Cacho J, Bendezu J, Neyra V, Bernal J, Gamboa D: **Asymptomatic malaria detection by PCR among a collateral native population in an endemic region at the Peruvian-Ecuadorian border.** *Conf Abstr [American J Trop Med Hyg* 2009:266.

2. Dacuma M, Hallett R, Dimalibot J, Ugaddan G, Yadao F, Notario W: **Asymptomatic Plasmodium vivax infections in southern Mindanao, Philippines: A challenge to malaria elimination**. *Conf Abstr [American J Trop Med Hyg* 2011:460.

3. Alencar F, Fernandes L, Buery J, Fux B, Falqueto A, Rezende H, Malafronte R: **Evaluation of asymptomatic carriers of Plasmodium sp in anendemic area covered by Atlantic forest, in the state of Espirito Santo, Brazil.** *Conf Abstr [American J Trop Med Hyg* 2011.

4. Soto-Calle V, Contreras J, Erhart A, Llanos-Cuentas A, Gamboa D, Speybroeck N, D’Alessandro U: **Malaria epidemiology in a sub-urban area of the peruvian amazon region**. *Conf Abstr [American J Trop Med Hyg* 2010:291.

5. Ali MSM, Yousif AGM, Mustafa MS, Ibrahim MH: **Evaluation of malaria parasite screening procedures among Sudanese blood donors.** *Clin Lab Sci J Am Soc Med Technol* 2005, **18**:69–73.

6. Manning P, Vo H, Vinetz J: **Malaria epidemiology in the illegal mining camps of madre de dios, peru.** *J Investig Med* 2013, **61**:184.

7. Khan W a, Galagan SR, Prue CS, Khyang J, Ahmed S, Ram M, Alam MS, Haq MZ, Akter J, Glass G, Norris DE, Shields T, Sack D a, Sullivan DJ, Nyunt MM: **Asymptomatic Plasmodium falciparum malaria in pregnant women in the Chittagong Hill Districts of Bangladesh.** *PLoS One* 2014, **9**:e98442.

8. Felger I, Tavul L, Narara A, Genton B, Alpers M, Beck H: **The use of the polymerase chain reaction for more sensitive detection of Plasmodium falciparum**. *P N G Med J* 1995, **38**:52–6.

9. Lee M a, Aw LT, Singh M: **A comparison of antigen dipstick assays with polymerase chain reaction (PCR) technique and blood film examination in the rapid diagnosis of malaria.** *Ann Acad Med Singapore* 1999, **28**:498–501.

10. Pacific Malaria Initiative Survey Group: **Malaria on isolated Melanesian islands prior to the initiation of malaria elimination activities.** *Malar J* 2010, **9**:218.

11. Khan W a, Sack D a, Ahmed S, Prue CS, Alam MS, Haque R, Khyang J, Ram M, Akter J, Nyunt MM, Norris D, Glass G, Shields T, Haq MZ, Cravioto A, Sullivan DJ: **Mapping hypoendemic, seasonal malaria in rural Bandarban, Bangladesh: a prospective surveillance.** *Malar J* 2011, **10**:124.

12. Rodulfo H, de Donato M, Quijada I, Peña A: **High prevalence of malaria infection in Amazonas State, Venezuela.** *Rev Inst Med Trop Sao Paulo* 2007, **49**:79–85.

13. Steenkeste N, Rogers WO, Okell L, Jeanne I, Incardona S, Duval L, Chy S, Hewitt S, Chou M, Socheat D, Babin F-X, Ariey F, Rogier C: **Sub-microscopic malaria cases and mixed malaria infection in a remote area of high malaria endemicity in Rattanakiri province, Cambodia: implication for malaria elimination.** *Malar J* 2010, **9**:108.

14. Andrade BB, Santos CJN, Camargo LM, Souza-Neto SM, Reis-Filho A, Clarêncio J, Mendonça VRR, Luz NF, Camargo EP, Barral A, Silva A a M, Barral-Netto M: **Hepatitis B infection is associated with asymptomatic malaria in the Brazilian Amazon.** *PLoS One* 2011, **6**:e19841.

15. Andrade BB, Rocha BC, Reis-Filho A, Camargo LMA, Tadei WP, Moreira LA, Barral A, Barral-Netto M: **Anti-Anopheles darlingi saliva antibodies as marker of Plasmodium vivax infection and clinical immunity in the Brazilian Amazon.** *Malar J* 2009, **8**:121.

16. Carrara VI, Lwin KM, Phyo AP, Ashley E, Wiladphaingern J, Sriprawat K, Rijken M, Boel M, McGready R, Proux S, Chu C, Singhasivanon P, White N, Nosten F: **Malaria burden and artemisinin resistance in the mobile and migrant population on the Thai-Myanmar border, 1999-2011: an observational study.** *PLoS Med* 2013, **10**:e1001398.

17. Das MK, Joshi H, Verma a, Singh SS, Adak T: **Malaria among the Jarawas, a primitive and isolated tribe on the Andaman islands, India.** *Ann Trop Med Parasitol* 2005, **99**:545–52.

18. De Almeida A, Rosário VE Do, Henriques G, Arez AP, Cravo P: **Plasmodium vivax in the Democratic Republic of East Timor: Parasite prevalence and antifolate resistance-associated mutations.** *Acta Trop* 2010, **115**:288–92.

19. Gomes ADC, Paula MB De, Duarte AMRDC, Lima M a, Malafronte RDS, Mucci LF, Gotlieb SLD, Natal D: **Epidemiological and ecological aspects related to malaria in the area of influence of the lake at Porto Primavera dam, in western São Paulo State, Brazil.** *Rev Inst Med Trop Sao Paulo* 2008, **50**:287–95.

20. Turki H, Zoghi S, Mehrizi a a, Zakeri S, Raeisi a, Khazan H, Haghdoost A: **Absence of asymptomatic malaria infection in endemic area of bashagard district, hormozgan province, iran.** *Iran J Parasitol* 2012, **7**:36–44.

21. Agudelo O, Arango E, Maestre A, Carmona-Fonseca J: **Prevalence of gestational, placental and congenital malaria in north-west Colombia.** *Malar J* 2013, **12**:341.

22. Mens PF, de Bes HM, Sondo P, Laochan N, Keereecharoen L, van Amerongen a, Flint J, Sak JRS, Proux S, Tinto H, Schallig HDFH: **Direct blood PCR in combination with nucleic acid lateral flow immunoassay for detection of Plasmodium species in settings where malaria is endemic.** *J Clin Microbiol* 2012, **50**:3520–5.

23. Arai M, Kunisada K, Kim H, Miyake H: **A Colorimetric DNA Diagnostic Method for Falciparum Malaria and Vivax Malaria : A Field Trial in the Solomon Islands**. *Nucleosides and Nucleotides* 1996, **15**:719–731.

24. Kritsiriwuthinan K, Ngrenngarmlert W: **Molecular screening of Plasmodium infections among migrant workers in Thailand.** *J Vector Borne Dis* 2011, **48**:214–8.

25. Kain KC, Brown a E, Mirabelli L, Webster HK: **Detection of Plasmodium vivax by polymerase chain reaction in a field study.** *J Infect Dis* 1993, **168**:1323–6.

26. Brownl AE: **Demonstration by the polymerase falciparum and P . vivax infections chain reaction of mixed Plasmodium undetected by conventional microscopy P-V**. 1992, **4**:609–612.

27. Contreras C, Donatro M, Rivas M, Rodulfo H, Mora R, Batista M, Marcano N: **Malaria seroprevalence in blood bank donors from endemic and non-endemic areas of Venezuela**. *Mem Inst Oswaldo Cruz* 2011, **106**:123–9.

28. Arroyo Sanchez M, Lima G, Inoue J, Carvalho M, Nascimento L, Sanchez A, Toniol C, De Jesus Nascimento M, Di Santi S: **Asymptomatic infections in blood donors harboring plasmodium detected by molecular and serological tools.** *Conf Abstr [Tropical Med Int Heal Conf* 2011:16.

29. Chaijaroenkul W, Wongchai T, Ruangweerayut R, Na-Bangchang K: **Evaluation of rapid diagnostics for Plasmodium falciparum and P. vivax in Mae Sot Malaria endemic area, Thailand.** *Korean J Parasitol* 2011, **49**:33–8.

30. Santana-Morales M a, Afonso-Lehmann RN, Quispe M a, Reyes F, Berzosa P, Benito A, Valladares B, Martinez-Carretero E: **Microscopy and molecular biology for the diagnosis and evaluation of malaria in a hospital in a rural area of Ethiopia.** *Malar J* 2012, **11**:199.

31. Monge-Maillo B, Lopez-Velez R: **Is screening for malaria necessary among asymptomatic refugees and immigrants coming from endemic countries?**. *Expert Rev Anti Infect Ther* 2011, **9**:521–4.

32. Lindo, F J, Bryce JH, Ducasse MB, Howitt C, Barrett DM, Morales JL, Ord R, Burke M, Chiodini PL, Sutherland CJ: **Plasmodium malariae in Haitian refugees, Jamaica**. *Emerg Infect Dis* 2007, **13**:931–933.

33. Matisz CE, Naidu P, Shokoples SE, Grice D, Krinke V, Brown SZ, Kowalewska-Grochowska K, Houston S, Yanow SK: **Post-arrival screening for malaria in asymptomatic refugees using real-time PCR.** *Am J Trop Med Hyg* 2011, **84**:161–5.

34. Ndao M, Bandyayera E, Kokoskin E, Gyorkos TW, Maclean JD, Ward BJ: **Comparison of Blood Smear , Antigen Detection , and Nested-PCR Methods for Screening Refugees from Regions Where Malaria Is Endemic after a Malaria Outbreak in Quebec , Canada**. *J Clin Microbiol* 2004, **42**:2694–2700.

35. Osman MMM, Nour BYM, Sedig MF, De Bes L, Babikir AM, Mohamedani A a, Mens PF: **Informed decision-making before changing to RDT: a comparison of microscopy, rapid diagnostic test and molecular techniques for the diagnosis and identification of malaria parasites in Kassala, eastern Sudan.** *Trop Med Int Health* 2010, **15**:1442–8.

36. Bernabeu M, Gomez-Perez GP, Sissoko S, Niambélé MB, Haibala AA, Sanz A, Théra M a, Fernandez-Becerra C, Traoré K, Alonso PL, Bassat Q, Del Portillo H a, Doumbo O: **Plasmodium vivax malaria in Mali: a study from three different regions.** *Malar J* 2012, **11**:405.

37. Rougemont M, Saanen M Van, Sahli R, Hinrikson HP, Bille J, Jaton K: **Detection of Four Plasmodium Species in Blood from Humans by 18S rRNA Gene Subunit-Based and Species-Specific Real-Time PCR Assays**. *J Clin Microbiol* 2004, **42**:5636–5643.

38. Bendezu J, Rosas A, Grande T, Rodriguez H, Llanos-Cuentas A, Escobedo J, Gamboa D: **Field evaluation of a rapid diagnostic test (Parascreen) for malaria diagnosis in the Peruvian Amazon.** *Malar J* 2010, **9**:154.

39. Iqbal J, Sher a, Hira PR, Al-Owaish R: **Comparison of the OptiMAL test with PCR for diagnosis of malaria in immigrants.** *J Clin Microbiol* 1999, **37**:3644–6.

40. Veron V, Simon S, Carme B: **Multiplex real-time PCR detection of P. falciparum, P. vivax and P. malariae in human blood samples.** *Exp Parasitol* 2009, **121**:346–51.

41. Pöschl B, Waneesorn J, Thekisoe O, Chutipongvivate S, Karanis P, Panagiotis K: **Comparative diagnosis of malaria infections by microscopy, nested PCR, and LAMP in northern Thailand.** *Am J Trop Med Hyg* 2010, **83**:56–60.

42. Heidari A, Keshavarz H: **Detection of low parasitemia malaria infections by nested PCR in the endemic region of Iran.** *Conf Abstr [Tropical Med Int Heal Conf* 2009:135.

43. Cnops L, Van Esbroeck M, Bottieau E, Jacobs J: **Giemsa-stained thick blood films as a source of DNA for Plasmodium species-specific real-time PCR.** *Malar J* 2010, **9**:370.

44. Andrade BB, Reis-Filho A, Barros AM, Souza-Neto SM, Nogueira LL, Fukutani KF, Camargo EP, Camargo LM a, Barral A, Duarte A, Barral-Netto M: **Towards a precise test for malaria diagnosis in the Brazilian Amazon: comparison among field microscopy, a rapid diagnostic test, nested PCR, and a computational expert system based on artificial neural networks.** *Malar J* 2010, **9**:117.

45. Cheng Z, Sun X, Yang Y, Wang H, Zheng Z: **A novel, sensitive assay for high-throughput molecular detection of plasmodia for active screening of malaria for elimination.** *J Clin Microbiol* 2013, **51**:125–30.

46. Proux S, Suwanarusk R, Barends M, Zwang J, Price RN, Leimanis M, Kiricharoen L, Laochan N, Russell B, Nosten F, Snounou G: **Considerations on the use of nucleic acid-based amplification for malaria parasite detection.** *Malar J* 2011, **10**:323.

47. Bharti PK, Chand SK, Singh MP, Mishra S, Shukla MM, Singh R, Singh N: **Emergence of a new focus of Plasmodium malariae in forest villages of district Balaghat, Central India: implications for the diagnosis of malaria and its control.** *Trop Med Int Health* 2013, **18**:12–7.

48. Eibach D, Traore B, Bouchrik M, Coulibaly B, Coulibaly N, Siby F, Bonnot G, Bienvenu A-L, Picot S: **Evaluation of the malaria rapid diagnostic test VIKIA malaria Ag Pf/Pan^TM^ in endemic and non-endemic settings.** *Malar J* 2013, **12**:188.

49. Parajuli K, Hanchana S, Inwomg M, Pukrittayakayamee S, Ghimire P: **Comparative evaluation of microscopy and polymerase chain reaction (PCR) for the diagnosis in suspected malaria patients of Nepal**. *Nepal Med Coll J* 2009, **11**:23–7.

50. Noteghpour M, Abed Khojasteh H, Keshavarz H, Hajjarah H, Edrissian G, Rahimpi A, Gobakhloo N: **Comparison of microscopical examination and semi-nested multiplex polymerase chain reaction in diagnosis of Plasmodium falciparum and Plasmodium vivax**. *East Medieterranean Heal J* 2011, **17**:51–5.

51. Zakeri S, Mamaghanim S, Mehrizi A, Shahsavari Z, Raeisi A, Arshi S, Dinparast-Djadid N: **Molecular evidence of mixed P.vivax and P.falciparum infections in northern Islamic Republic of Iran**. *East Medieterranean Heal J* 2004, **10**:336–42.

52. Zakeri S, Najafabadi ST, Zare A, Djadid ND: **Detection of malaria parasites by nested PCR in south-eastern, Iran: evidence of highly mixed infections in Chahbahar district.** *Malar J* 2002, **1**:2.

53. Yan J, Li N, Wei X, Li P, Zhao Z, Wang L, Li S, Li X, Wang Y, Li S, Yang Z, Zheng B, Zhou G, Yan G, Cui L, Cao Y, Fan Q: **Performance of two rapid diagnostic tests for malaria diagnosis at the China-Myanmar border area.** *Malar J* 2013, **12**:73.

54. Woldearegai TG, Kremsner PG, Kun JFJ, Mordmüller B: **Plasmodium vivax malaria in Duffy-negative individuals from Ethiopia.** *Trans R Soc Trop Med Hyg* 2013, **107**:328–31.

55. Johnston SP, Pieniazek NJ, Xayavong M V, Slemenda SB, Wilkins PP, Silva AJ: **PCR as a Confirmatory Technique for Laboratory Diagnosis of Malaria**. *J Clin Microbiol* 2006, **44**:1087–1089.

56. Woyessa A, Deressa W, Ali A, Lindtjørn B: **Evaluation of CareStart^TM^ malaria Pf/Pv combo test for Plasmodium falciparum and Plasmodium vivax malaria diagnosis in Butajira area, south-central Ethiopia.** *Malar J* 2013, **12**:218.

57. Kim T-S, Kim H-H, Lee S-S, Na B-K, Lin K, Cho S-H, Kang Y-J, Kim D-K, Sohn Y, Kim H, Lee H-W: **Prevalence of Plasmodium vivax VK210 and VK247 subtype in Myanmar.** *Malar J* 2010, **9**:195.

58. Maltha J, Gillet P, Bottieau E, Cnops L, van Esbroeck M, Jacobs J: **Evaluation of a rapid diagnostic test (CareStart Malaria HRP-2/pLDH (Pf/pan) Combo Test) for the diagnosis of malaria in a reference setting.** *Malar J* 2010, **9**:171.

59. Boonma P, Christensen PR, Suwanarusk R, Price RN, Russell B, Lek-Uthai U: **Comparison of three molecular methods for the detection and speciation of Plasmodium vivax and Plasmodium falciparum.** *Malar J* 2007, **6**:124.

60. Postigo M, Mendoza-Leon A, Perez H: **Malaria diagnosis by the polymerase eastern Venezuela Milagros chain reaction : a field study in south-Malaria diagnosis by the polymerase chain reaction: a field study in south-eastern Venezuela**. *Trans R Soc Trop Med Hyg* 1998, **92**:509–11.

61. Joveen-Neoh WF, Chong KL, Wong CMVL, Lau TY: **Incidence of malaria in the interior division of sabah, malaysian borneo, based on nested PCR.** *J Parasitol Res* 2011, **2011**:104284.

62. Auma MA, Siedner MJ, Nyehangane D, Nalusaji A, Nakaye M, Mwanga-amumpaire J, Muhindo R, Wilson LA, Ii YB, Moore CC: **Malaria is an uncommon cause of adult sepsis in south-Western Uganda**. *Malar J* 2013, **12**:1–9.

63. Mohon AN, Elahi R, Podder MP, Mohiuddin K, Hossain MS, Khan W a, Haque R, Alam MS: **Evaluation of the OnSite (Pf/Pan) rapid diagnostic test for diagnosis of clinical malaria.** *Malar J* 2012, **11**:415.

64. Gargate M, Drago M, Vilares A, Monteiro L, Correia M, Bernardino L, Angelo H: **Development of a real time PCR assay for differentiation of Plasmodium species in a pediatric sample from Luanda.** *Conf Abstr [Tropical Med Int Heal Conf* 2009:14.

65. Orjih AU, Cherian P, AlFadhli S: **Microscopic detection of mixed malarial infections: improvement by saponin hemolysis.** *Med Princ Pract* 2008, **17**:458–63.

66. Snounou G, Viriyakosol S, Zhu XP, Jarra W, Pinheiro L, do Rosario VE, Thaithong S, Brown KN: **High sensitivity of detection of human malaria parasites by the use of nested polymerase chain reaction.** *Mol Biochem Parasitol* 1993, **61**:315–20.

67. Hwang S-Y, Kim S-H, Lee G-Y, Hang VTT, Moon C-S, Shin JH, Koo W-L, Kim S-Y, Park H-J, Park H-O, Kho W-G: **A novel real-time PCR assay for the detection of Plasmodium falciparum and Plasmodium vivax malaria in low parasitized individuals.** *Acta Trop* 2011, **120**:40–5.

68. Mohapatra PK, Prakash A, Bhattacharyya DR, Goswami BK, Ahmed A, Sarmah B, Mahanta J: **Detection & molecular confirmation of a focus of Plasmodium malariae in Arunachal Pradesh, India.** *Indian J Med Res* 2008, **128**:52–6.

69. Parkes R, Lo T, Wong Q, Isaac-Renton J, Byrne S: **Comparison of nested polymerase chain reaction-restriction fragment length polymorphism method, the PATH antigen detection method, and microscopy for the detection and identification of malaria parasites**. *Can J Microbiol* 2001, **47**:903–7.

70. Fuehrer H-P, Starzengruber P, Swoboda P, Khan WA, Matt J, Ley B, Thriemer K, Haque R, Yunus E Bin, Hossain SM, Walochnik J, Noedl H: **Indigenous Plasmodium ovale malaria in Bangladesh.** *Am J Trop Med Hyg* 2010, **83**:75–8.

71. Kuamsab N, Putaporntip C, Pattanawong U, Jongwutiwes S: **Simultaneous detection of Plasmodium vivax and Plasmodium falciparum gametocytes in clinical isolates by multiplex-nested RT-PCR.** *Malar J* 2012, **11**:190.

72. Pakalapati D, Garg S, Middha S, Kochar A, Subudhi A, Arunachalam, BP, Kochar S, Saxena V, Pareek R, Acharya J, Kochar D, Das A: **Comparative evaluation of microscopy, OptiMAL and 18S rRNA gene based multiplex PCR for detection of Plasmodium falciparum & Plasmodium vivax from field isolates of Bikaner, India**. *Asia Pacific J Trop Med* 2013, **13**:346–51.

73. Barman D, Mirdha B, Samantray J, Kironde F, Kabra S, Guleria R: **Evaluation of quantitative buffy coat (QBC) assay and polymerase chain reaction (PCR) for diagnosis of malaria**. *J Commun Dis* 2003, **35**:170–81.

74. Mens PF, van Amerongen A, Sawa P, Kager P a, Schallig HDFH: **Molecular diagnosis of malaria in the field: development of a novel 1-step nucleic acid lateral flow immunoassay for the detection of all 4 human Plasmodium spp. and its evaluation in Mbita, Kenya.** *Diagn Microbiol Infect Dis* 2008, **61**:421–7.

75. Barker R, Banchongaksorn T, Courval J, Suwomkerd W, Rimwungtragoon K, Wirth D: **Plasmodium falciparum and P.vivax: Factors affecting Sensitivity and Specificity of PCR-based Diagnosis of Malaria**. *Exp Parasitol* 994, **79**:41–49.

76. Lekweiry KM, Abdallahi MO, Ba H, Arnathau C, Durand P, Trape J, Ould A, Salem M: **Preliminary study of malaria incidence in Nouakchott , Mauritania**. *Malar J* 2009, **7**:1–7.

77. Noedl H, Yingyuen K, Laoboonchai A, Fukuda M, Sirichaisinthop J, Miller RS: **Sensitivity and specificity of an antigen detection elisa for malaria diagnosis**. *Am Jouranl Trop Med Hyg* 2006, **75**:1205–1208.

78. Pakalapati D, Garg S, Middha S, Acharya J, Subudhi AK, Boopathi AP, Saxena V, Kochar SK, Kochar DK, Das A: **Development and evaluation of a 28S rRNA gene-based nested PCR assay for P. falciparum and P. vivax.** *Pathog Glob Health* 2013, **107**:180–8.

79. Haghdoost A-A, Mazhari S, Bahadini K: **Comparing the results of light microscopy with the results of PCR method in the diagnosis of Plasmodium vivax.** *J Vector Borne Dis* 2006, **43**:53–7.

80. Laoboonchai a, Kawamoto F, Thanoosingha N, Kojima S, Scott Miller RR, Kain KC, Wongsrichanalai C: **PCR-based ELISA technique for malaria diagnosis of specimens from Thailand.** *Trop Med Int Health* 2001, **6**:458–62.

81. Tao Z-Y, Zhou H-Y, Xia H, Xu S, Zhu H-W, Culleton RL, Han E-T, Lu F, Fang Q, Gu Y-P, Liu Y-B, Zhu G-D, Wang W-M, Li J-L, Cao J, Gao Q: **Adaptation of a visualized loop-mediated isothermal amplification technique for field detection of Plasmodium vivax infection.** *Parasit Vectors* 2011, **4**:115.

82. A-Elgayoum SME, El-Rayah E, Giha H a: **In areas of low transmission, is the presumptive treatment of febrile but bloodsmear-negative patients for malaria validated by the results of PCR-based testing?**. *Ann Trop Med Parasitol* 2010, **104**:573–81.

83. Alam MS, Mohon AN, Mustafa S, Khan WA, Islam N, Karim MJ, Khanum H, Sullivan DJ, Haque R: **Real-time PCR assay and rapid diagnostic tests for the diagnosis of clinically suspected malaria patients in Bangladesh.** *Malar J* 2011, **10**:175.

84. Genc A, Eroglu F, Koltas IS: **Detection of Plasmodium vivax by nested PCR and real-time PCR.** *Korean J Parasitol* 2010, **48**:99–103.

85. Ebrahimzadeh a, Fouladi B, Fazaeli a: **High rate of detection of mixed infections of Plasmodium vivax and Plasmodium falciparum in South-East of Iran, using nested PCR.** *Parasitol Int* 2007, **56**:61–4.

86. Lekweiry KM, Abdallahi MO, Ba H, Arnathau C, Durand P, Trape J-F, Salem AOM: **Preliminary study of malaria incidence in Nouakchott, Mauritania.** *Malar J* 2009, **8**:92.

87. Laserson K, Petralanda I, Hamlin D, Almera R, Fuentes M, Carrasquel A, Barker R: **Use of the polymerase chain reaction to directly detect malaria parasites in blood samples from the Venezuelan Amazon**. *Am Jouranl Trop Med Hyg* 1994, **50**:169–80.

88. Swan H, Sloan L, Muyombwe A, Chavalitshewinkoon-Petmitr P, Krudsood S, Leowattana W, Wilairatana P, Looareesuwan S, Rosenblatt J: **Evaluation of a real-time polymerase chain reaction assay for the diagnosis of malaria in patients from Thailand.** *Am J Trop Med Hyg* 2005, **73**:850–4.

89. Baltzell K a, Shakely D, Hsiang M, Kemere J, Ali AS, Björkman A, Mårtensson A, Omar R, Elfving K, Msellem M, Aydin-Schmidt B, Rosenthal PJ, Greenhouse B: **Prevalence of PCR detectable malaria infection among febrile patients with a negative Plasmodium falciparum specific rapid diagnostic test in Zanzibar.** *Am J Trop Med Hyg* 2013, **88**:289–91.

90. Singh N, Shukla MM, Shukla MK, Mehra RK, Sharma S, Bharti PK, Singh MP, Singh A, Gunasekar A: **Field and laboratory comparative evaluation of rapid malaria diagnostic tests versus traditional and molecular techniques in India.** *Malar J* 2010, **9**:191.

91. Zhou X, Li S-G, Chen S-B, Wang J-Z, Xu B, Zhou H-J, Ge H-XZ, Chen J-H, Hu W: **Co-infections with Babesia microti and Plasmodium parasites along the China-Myanmar border.** *Infect Dis poverty* 2013, **2**:24.

92. Alemu A, Fuehrer H-P, Getnet G, Tessema B, Noedl H: **Plasmodium ovale curtisi and Plasmodium ovale wallikeri in North-West Ethiopia.** *Malar J* 2013, **12**:346.

93. Puri B, Mehta P, Ingole NA, Prasad P, Mathure T: **Laboratory tests for malaria: a diagnostic conundrum?**. *South Arfrican Med J* 2013, **103**:625–7.

94. Mekonnen SK, Aseffa A, Medhin G, Berhe N, Velavan TP: **Re-evaluation of microscopy confirmed Plasmodium falciparum and Plasmodium vivax malaria by nested PCR detection in southern Ethiopia.** *Malar J* 2014, **13**:48.

95. Herrera S, Vallejo AF, Quintero JP, Arévalo-Herrera M, Cancino M, Ferro S: **Field evaluation of an automated RDT reader and data management device for Plasmodium falciparum/Plasmodium vivax malaria in endemic areas of Colombia.** *Malar J* 2014, **13**:87.

96. Ghayour Najafabadi Z, Oormazdi H, Akhlaghi L, Meamar AR, Nateghpour M, Farivar L, Razmjou E: **Detection of Plasmodium vivax and Plasmodium falciparum DNA in human saliva and urine: loop-mediated isothermal amplification for malaria diagnosis.** *Acta Trop* 2014, **136**:44–9.

97. Ghayour Najafabadi Z, Oormazdi H, Akhlaghi L, Meamar AR, Raeisi A, Rampisheh Z, Nateghpour M, Razmjou E: **Mitochondrial PCR-based malaria detection in saliva and urine of symptomatic patients.** *Trans R Soc Trop Med Hyg* 2014, **108**:358–62.

98. Singh R, Singh DP, Gupta R, Savargaonkar D, Singh OP, Nanda N, Bhatt RM, Valecha N: **Comparison of three PCR-based assays for the non-invasive diagnosis of malaria: detection of Plasmodium parasites in blood and saliva.** *Eur J Clin Microbiol Infect Dis* 2014, **33**:1631–9.

99. Alemu A, Fuehrer H-P, Getnet G, Kassu A, Getie S, Noedl H: **Comparison of Giemsa microscopy with nested PCR for the diagnosis of malaria in North Gondar, north-west Ethiopia.** *Malar J* 2014, **13**:174.

100. Zhou X, Huang J-L, Njuabe MT, Li S-G, Chen J-H, Zhou X-N: **A molecular survey of febrile cases in malaria-endemic areas along China-Myanmar border in Yunnan province, People’s Republic of China.** *Parasite* 2014, **21**:27.

101. Kim J-Y, Goo Y-K, Ji S-Y, Shin H-I, Han E-T, Hong Y, Chung D-I, Cho S-H, Lee W-J: **Development and efficacy of real-time PCR in the diagnosis of vivax malaria using field samples in the Republic of Korea.** *PLoS One* 2014, **9**:e105871.

102. Shahzadi S, Akhtar T, Hanif A, Sahar S: **Molecular detection of malaria in South Punjab with higher proportion of mixed infections**. *Iran J Parasitol* 2014, **9**:37–43.

103. Shahbazi A, Farhadi P, Yerian M: **Detection of Asymptomatic Carriers of Plasmodium vivax among Treated Patients by Nested PCR Method in Minab, Rudan and Bashagard, Iran**. *Iran J …* 2013, **8**:586–592.

104. Ayala E, Lescano AG, Gilman RH, Calderón M, Pinedo V V, Terry H, Cabrera L, Vinetz JM: **Polymerase chain reaction and molecular genotyping to monitor parasitological response to anti-malarial chemotherapy in the Peruvian Amazon.** *Am J Trop Med Hyg* 2006, **74**:546–53.

105. Tirasophon W, Rajkulchai P, Wilairat P, Boonsaeng V, Panyim S: **A highly sensitive, rapid, and simple polymerase chain reaction-based method to detect human malaria (Plasmodium falciparum and Plasmodium vivax) in blood samples**. *Am Jouranl Trop Med Hyg* 1994, **51**:308–313.

106. Han E-T, Watanabe R, Sattabongkot J, Khuntirat B, Sirichaisinthop J, Iriko H, Jin L, Takeo S, Tsuboi T: **Detection of four Plasmodium species by genus- and species-specific loop-mediated isothermal amplification for clinical diagnosis.** *J Clin Microbiol* 2007, **45**:2521–8.

107. Kho W-G, Chung J-Y, Sim E-J, Kim M-Y, Kim D-W, Jongwutiwes S, Tanabe K: **A multiplex polymerase chain reaction for a differential diagnosis of Plasmodium falciparum and Plasmodium vivax**. *Parasitol Int* 2003, **52**:229–236.

108. Zakeri S, Kakar Q, Ghasemi F, Raeisi A, Butt W, Safi N, Afsharpad M, Memon MS, Gholizadeh S, Salehi M, Atta H, Zamani G, Djadid ND: **Detection of mixed Plasmodium falciparum & P.vivax infections by nested-PCR in Pakistan, Iran and Afghanistan**. *Indian J Med Res* 2010(July):31–35.

109. Gupta B, Gupta P, Sharma A, Singh V, Dash AP, Das A: **High proportion of mixed-species Plasmodium infections in India revealed by PCR diagnostic assay.** *Trop Med Int Health* 2010, **15**:819–24.

110. Van den Eede P, Soto-Calle VE, Delgado C, Gamboa D, Grande T, Rodriguez H, Llanos-Cuentas A, Anné J, D’Alessandro U, Erhart A: **Plasmodium vivax sub-patent infections after radical treatment are common in Peruvian patients: results of a 1-year prospective cohort study.** *PLoS One* 2011, **6**:e16257.

111. Farooq U, Malla N, Dubey ML: **Polymorphism in merozoite surface protein-1 gene in north & northwest Indian field isolates of Plasmodium vivax.** *Indian J Med Res* 2009, **130**:736–41.

112. Reller ME, Chen WH, Dalton J, Lichay M a, Dumler JS: **Multiplex 5’ Nuclease Quantitative Real-Time PCR for Clinical Diagnosis of Malaria and Species-Level Identification and Epidemiologic Evaluation of Malaria-Causing Parasites, Including Plasmodium knowlesi.** *J Clin Microbiol* 2013, **51**:2931–8.

113. Putaporntip C, Buppan P, Jongwutiwes S: **Improved performance with saliva and urine as alternative DNA sources for malaria diagnosis by mitochondrial DNA-based PCR assays.** *Clin Microbiol Infect* 2011, **17**:1484–91.

114. Dormond L, Jaton-Ogay K, de Vallière S, Genton B, Bille J, Greub G: **Multiplex real-time PCR for the diagnosis of malaria: correlation with microscopy.** *Clin Microbiol Infect* 2011, **17**:469–75.

115. Ramírez-Olivencia G, Rubio JM, Rivas P, Subirats M, Herrero MD, Lago M, Puente S: **Imported submicroscopic malaria in Madrid.** *Malar J* 2012, **11**:324.

116. Congpuong K, Pedro J, Bualombai P, Kangchaingone Y: **Mixed-species malaria infection in high transmission areas of Thailand**. *Asian Biomed* 2008, **2**:117–121.

117. Albuquerque SRL, Cavalcante FDO, Sanguino EC, Tezza L, Chacon F, Castilho L, dos Santos MC: **FY polymorphisms and vivax malaria in inhabitants of Amazonas State, Brazil.** *Parasitol Res* 2010, **106**:1049–53.

118. Lek D, Rogers W, Ariey F, Bruce J, Meek S, Babu S, Duong S: **Nationwide prevalence of malaria in Cambodia in 2007: Comparison of microscopy and PCR**. *Conf Abstr [American J Trop Med Hyg* 2010:47.

119. Owusu-Agyei S, Smith T, Beck H-P, Amenga-Etego L, Felger I: **Molecular epidemiology of Plasmodium falciparum infections among asymptomatic inhabitants of a holoendemic malarious area in northern Ghana.** *Trop Med Int Health* 2002, **7**:421–8.

120. Kalayjian BC, Malhotra I, Mungai P, Holding P, King CL: **Marked decline in malaria prevalence among pregnant women and their offspring from 1996 to 2010 on the south Kenyan Coast.** *Am J Trop Med Hyg* 2013, **89**:1129–34.

121. Sattabongkot J, Tsuboi T, Han E-T, Bantuchai S, Buates S: **Loop-mediated isothermal amplification assay for rapid diagnosis of malaria infections in an area of endemicity in Thailand.** *J Clin Microbiol* 2014, **52**:1471–7.

122. Noor AM, Mohamed MB, Mugyenyi CK, Osman M a, Guessod HH, Kabaria CW, Ahmed I a, Nyonda M, Cook J, Drakeley CJ, Mackinnon MJ, Snow RW: **Establishing the extent of malaria transmission and challenges facing pre-elimination in the Republic of Djibouti.** *BMC Infect Dis* 2011, **11**:121.

123. Ataka Y, Ohtsuka R, Inaoka T, Kawabata M, Ohashi J, Matsushita M, Tokunaga K, Kano S, Suzuki M: **Variation in Malaria Endemicity in Relation to Microenvironmental Conditions in the Admiralty Islands, Papua New Guinea**. *Asia-Pacific J Public Heal* 2001, **13**:85–90.

124. Cucunubá ZM, Guerra ÁP, Rivera JA, Nicholls RS: **Comparison of asymptomatic Plasmodium spp. infection in two malaria-endemic Colombian locations.** *Trans R Soc Trop Med Hyg* 2013, **107**:129–36.

125. Canier L, Khim N, Kim S, Sluydts V, Heng S, Dourng D, Eam R, Chy S, Khean C, Loch K, Ken M, Lim H, Siv S, Tho S, Masse-Navette P, Gryseels C, Uk S, Van Roey K, Grietens KP, Sokny M, Thavrin B, Chuor CM, Deubel V, Durnez L, Coosemans M, Ménard D: **An innovative tool for moving malaria PCR detection of parasite reservoir into the field.** *Malar J* 2013, **12**:405.

126. Mendes C, Dias F, Figueiredo J, Mora VG, Cano J, de Sousa B, do Rosário VE, Benito A, Berzosa P, Arez AP: **Duffy negative antigen is no longer a barrier to Plasmodium vivax--molecular evidences from the African West Coast (Angola and Equatorial Guinea).** *PLoS Negl Trop Dis* 2011, **5**:e1192.

127. Fru-Cho J, Bumah V V, Safeukui I, Nkuo-Akenji T, Titanji VPK, Haldar K: **Molecular typing reveals substantial Plasmodium vivax infection in asymptomatic adults in a rural area of Cameroon.** *Malar J* 2014, **13**:170.

128. Herdiana H, Fuad A, Asih PB, Zubaedah S, Arisanti RR, Syafruddin D, Kusnanto H, Sumiwi ME, Yuniarti T, Imran A, Rahmadyani R, Yani M, Kusriastuti R, Tarmizi SN, Laihad FJ, Hawley W a: **Progress towards malaria elimination in Sabang Municipality, Aceh, Indonesia.** *Malar J* 2013, **12**:42.

129. Cucunubá ZM, Guerra A, Rahirant S, Rivera J, Cortes L, Nicholls R: **Asymptomatic Plasmodium spp. infection in Tierralta, Colombia**. *Mem Inst Oswaldo Cruz* 2008, **103**:668–73.

130. Da Silva-Nunes M, Codeço CT, Malafronte RS, da Silva NS, Juncansen C, Muniz PT, Ferreira MU: **Malaria on the Amazonian frontier: transmission dynamics, risk factors, spatial distribution, and prospects for control.** *Am J Trop Med Hyg* 2008, **79**:624–35.

131. Kasehagen LJ, Mueller I, Kiniboro B, Bockarie MJ, Reeder JC, Kazura JW, Kastens W, McNamara DT, King CH, Whalen CC, Zimmerman P a: **Reduced Plasmodium vivax erythrocyte infection in PNG Duffy-negative heterozygotes.** *PLoS One* 2007, **2**:e336.

132. Thang ND, Erhart A, Hung LX, Thuan LK, Xa NX, Thanh NN, Ky P Van, Coosemans M, Speybroeck N, Alessandro UD: **Rapid decrease of malaria morbidity following the introduction of community-based monitoring in a rural area of central Vietnam**. *Malar J* 2009, **8**:1–10.

133. Scopel KKG, Fontes CJF, Nunes AC, Horta MF, Braga EM: **Low sensitivity of nested PCR using Plasmodium DNA extracted from stained thick blood smears: an epidemiological retrospective study among subjects with low parasitaemia in an endemic area of the Brazilian Amazon region.** *Malar J* 2004, **3**:8.

134. Culleton RL, Mita T, Ndounga M, Unger H, Cravo PVL, Paganotti GM, Takahashi N, Kaneko A, Eto H, Tinto H, Karema C, D’Alessandro U, do Rosário V, Kobayakawa T, Ntoumi F, Carter R, Tanabe K: **Failure to detect Plasmodium vivax in West and Central Africa by PCR species typing.** *Malar J* 2008, **7**:174.

135. Harris I, Sharrock WW, Bain LM, Gray K-A, Bobogare A, Boaz L, Lilley K, Krause D, Vallely A, Johnson M-L, Gatton ML, Shanks GD, Cheng Q: **A large proportion of asymptomatic Plasmodium infections with low and sub-microscopic parasite densities in the low transmission setting of Temotu Province, Solomon Islands: challenges for malaria diagnostics in an elimination setting.** *Malar J* 2010, **9**:254.

136. Van Den Eede P, Van H, Van Overmeir C, Vythilingam I, Ngo Duc T, Nguyen Xuan X, Le Xuan H, Nguyen Manh H, Anne J, D’Alessandro U, Erhart A: **Human Plasmodium knowlesi infections in Central Vietnam.** *Conf Abstr [Tropical Med Int Heal Conf* 2009:14.

137. Win TT, Lin K, Mizuno S, Zhou M, Liu Q, Ferreira MU, Tantular IS, Kojima S, Ishii a, Kawamoto F: **Wide distribution of Plasmodium ovale in Myanmar.** *Trop Med Int Health* 2002, **7**:231–9.

138. Bharti AR, Patra KP, Chuquiyauri R, Kosek M, Gilman RH, Llanos-cuentas A, Vinetz JM: **Short Report : Polymerase Chain Reaction Detection of Plasmodium vivax and Plasmodium falciparum DNA from Stored Serum Samples : Implications for Retrospective Diagnosis of Malaria**. *Am Jouranl Trop Med Hyg* 2007, **77**:444–446.

139. Quintana M, Piper R, Boling HL, Makler M, Sherman C, Gill E, Fernandez E, Martin S: **Malaria diagnosis by dipstick assay in a Honduran population with coendemic Plasmodium falciparum and Plasmodium vivax.** *Am J Trop Med Hyg* 1998, **59**:868–71.

140. Dawoud H., Ageely H., Heiba A.: **Evaluation of real-time polymerase chain reaction assay for the diagnosis of malaria in patients from Jazan area, Saudi Arabia**. *J Egypt Soc Parasitol* 2008, **38**:339–350.

141. Mahajan B, Zheng H, Pham PT, Sedegah MY, Majam VF, Akolkar N, Rios M, Ankrah I, Madjitey P, Amoah G, Addison E, Quakyi I a, Kumar S: **Polymerase chain reaction-based tests for pan-species and species-specific detection of human Plasmodium parasites.** *Transfusion* 2012, **52**:1949–56.

142. Curado I, Dos Santos Malafronte R, de Castro Duarte AMR, Kirchgatter K, Branquinho MS, Bianchi Galati EA: **Malaria epidemiology in low-endemicity areas of the Atlantic Forest in the Vale do Ribeira, São Paulo, Brazil.** *Acta Trop* 2006, **100**:54–62.
